# Differential Postnatal Expression of Neuronal Maturation Markers in the Dentate Gyrus of Mice and Rats

**DOI:** 10.3389/fnana.2017.00104

**Published:** 2017-11-14

**Authors:** Tijana Radic, Lara Frieß, Aruvi Vijikumar, Tassilo Jungenitz, Thomas Deller, Stephan W. Schwarzacher

**Affiliations:** Institute of Clinical Neuroanatomy, Neuroscience Center, Goethe University, Frankfurt am Main, Germany

**Keywords:** postnatal neurogenesis, adult neurogenesis, dentate gyrus, doublecortin, calbindin, rat, mouse

## Abstract

The dentate gyrus (DG) is a unique structure of the hippocampus that is distinguished by ongoing neurogenesis throughout the lifetime of an organism. The development of the DG, which begins during late gestation and continues during the postnatal period, comprises the structural formation of the DG as well as the establishment of the adult neurogenic niche in the subgranular zone (SGZ). We investigated the time course of postnatal maturation of the DG in male C57BL/6J mice and male Sprague-Dawley rats based on the distribution patterns of the immature neuronal marker doublecortin (DCX) and a marker for mature neurons, calbindin (CB). Our findings demonstrate that the postnatal DG is marked by a substantial maturation with a high number of DCX-positive granule cells (GCs) during the first two postnatal weeks followed by a progression toward more mature patterns and increasing numbers of CB-positive GCs within the subsequent 2 weeks. The most substantial shift in maturation of the GC population took place between P7 and P14 in both mice and rats, when young, immature DCX-positive GCs became confined to the innermost part of the GC layer (GCL), indicative of the formation of the SGZ. These results suggest that the first month of postnatal development represents an important transition phase during which DG neurogenesis and the maturation course of the GC population becomes analogous to the process of adult neurogenesis. Therefore, the postnatal DG could serve as an attractive model for studying a growing and functionally maturing neural network. Direct comparisons between mice and rats revealed that the transition from immature DCX-positive to mature CB-positive GCs occurs more rapidly in the rat by approximately 4–6 days. The remarkable species difference in the speed of maturation on the GC population level may have important implications for developmental and neurogenesis research in different rodent species and strains.

## Introduction

The process of hippocampal adult neurogenesis has been the subject of extensive study in different types of mammals, including humans, due to its pertinence to hippocampus-related cognitive functions and its potential use for regenerative therapy and brain repair (Zhao et al., 2008; Spalding et al., 2013; Kempermann et al., 2015; Gonçalves et al., 2016). The dentate gyrus (DG) of the mammalian hippocampus is a region in the brain in which neurogenesis persists throughout life. The rodent DG has unique developmental characteristics, including the fact that the vast majority, i.e., 85% of the principal neurons, the granule cells (GCs), are generated postnatally (Angevine, 1965; Schlessinger et al., 1975; Altman and Bayer, 1990a). Embryonic DG development follows a similar sequence of events in mice and rats, albeit with a different time course, as the appearance of first GCs begins at embryonic day 10 (E10) in mice and as late as E16 in rats (Angevine, 1965; Altman and Bayer, 1990a, b). Postnatally, neural stem cells (NSCs) continue to produce neurons that are added to the supra- and infrapyramidal blades of the embryonically formed GC layer (GCL; Altman and Bayer, 1990a, b; Pleasure et al., 2000; Li and Pleasure, 2005; Li et al., 2009; Hodge et al., 2013) and undergo a reorganization to establish the neurogenic niche of the subgranular zone (SGZ) that provides a unique environment for the maintenance of NSCs throughout adulthood (Altman and Das, 1965; Altman and Bayer, 1990a; Seri et al., 2004; Fuentealba et al., 2012; Bond et al., 2015).

Overall, the postnatal phase of GC development appears to be an important transitory period between embryonic formation and continuation toward neurogenesis in a matured network and thus could serve as an attractive model for studying the influences of a growing and functionally maturing network on the postnatal establishment of the neurogenic niche in the SGZ. Although in general adult-born GCs appear to largely follow the development of perinatally generated GCs (Espósito et al., 2005; Laplagne et al., 2006; Piatti et al., 2006), the time course of postnatal DG maturation to the adult state is not established in detail. Recently, it has been suggested that the formation of the distinct “adult” neurogenic niche in the SGZ occurs as an extension of DG development postnatally between P7 and P14 in mice (Nicola et al., 2015). As embryonic and early postnatal neurogenesis presumably proceed at a faster rate than the adult (Zhao et al., 2006), a deceleration of GC maturation may be postulated during the early postnatal phase approaching the adult state.

In the current work, we characterized the time course of postnatal maturation of the GC population with a number of established molecular markers (Kempermann et al., 2004, 2015; Ming and Song, 2011) in the DG from an early postnatal period (P7) until animals reached sexual maturity (P42). Since it is well-known that the time course of adult-born GC development differs in distinct rodent species (Snyder et al., 2009), postnatal DG development was investigated in both mice and rats, and species-dependent differences were analyzed. Our findings reveal that the shift of the GC population toward a mature neuronal phenotype was most pronounced between P7 and P14 in both species. However, the course of maturation in the GC population was faster in rats compared to mice by 4–6 days. These results show that during the postnatal development of the rodent DG, the most prominent transition to GC population maturation occurred within the first 4 weeks of development and demonstrate important developmental differences between mice and rats that ought to be considered in future studies using different rodent species.

## Materials and Methods

### Animals

Male Sprague-Dawley rats (P7: *n* = 3; P14: *n* = 3; P21: *n* = 4; P28: *n* = 3; P35: *n* = 3; and P42: *n* = 4) and male C57BL/6J mice (P7: *n* = 4; P14: *n* = 3; P21: *n* = 3; P28: *n* = 5; P35: *n* = 5; and P42: *n* = 3) were bred and housed at the animal facility of the Goethe-University hospital Frankfurt/Main, Germany. Animals were housed under standard conditions in a 12 h dark/light cycle with food and water available *ad libitum*. Animal care and experimental procedures were performed in agreement with the German law on the use of laboratory animals (animal welfare act; TierSchG; §4 par 3) and approved by the animal welfare officer of Goethe University, Frankfurt Faculty of Medicine.

### Immunohistochemistry

Animals were killed with an overdose of isoflurane and transcardially perfused with 0.9% NaCl followed by 4% paraformaldehyde (PFA) in 0.1 M phosphate buffered saline (PBS). Brains were removed, postfixed in 4% PFA overnight at 4°C, and serially sliced into 50 μm thick frontal sections with a vibratome (Leica VT1000S) and stored in cryoprotectant solution containing 30% ethylene glycol, 25% glycerol and 0.01% NaN_3_ in 0.1 M PBS at −20°C. For immunohistochemistry, sections were collected from the first section in which both the supra- and infrapyramidal blades of the dorsal DG were clearly visible. From there, every 6th section within the first millimeter was selected. For 7-day-old brains, every 5th section was selected. Free floating sections were first washed three times for 5 min in TRIS-buffered saline (TBS; pH 7.40) + 0.01% NaN_3_. Subsequently, sections were incubated in a blocking solution containing 5% bovine serum albumin and 0.5% Triton X-100 for 1 h at room temperature. Next, sections were placed in primary antibody solution containing the relevant antibodies in TBS + 0.01% NaN_3,_ 0.1% Triton X-100, and 1% BSA overnight at room temperature. The following primary antibodies were used: anti-calbindin (anti-CB, mouse, monoclonal, 1:500, Swant), anti-Prospero-related homeobox 1 gene (Prox1, rabbit, polyclonal, 1:1000, ReliaTech), and anti-doublecortin (anti-DCX, goat, polyclonal, 1:500, Santa Cruz). Sections were washed in TBS + 0.01% NaN_3_ three times for 5 min and then incubated with secondary antibodies that were conjugated with fluorescent dye (Alexa Fluor donkey anti mouse 488, donkey anti rabbit 568 and donkey anti goat 633, 1:1000, Molecular Probes) in TBS + 0.01% NaN_3,_ 0.1% Triton X-100, and 1% BSA overnight at room temperature. Next, sections were washed three times in TBS + 0.01% NaN_3_ and mounted with DAKO fluorescent mounting medium.

### Imaging and Analysis

High resolution (1024 × 1024 pixel) confocal images of histological frontal sections (three sections per animal) were obtained with a confocal laser scanning microscope (Nikon Eclipse 80i) equipped with a camera (Nikon D-Eclipse C1) using a 40× oil immersion lens (N.A. 1.3) and the software EZ-C1 3.60. In each section, three adjacent, non-overlapping regions of interest (medial, middle and lateral), were chosen for imaging along the suprapyramidal blade of the dorsal (septal) DG (bilaterally) starting directly laterally from the crest where the supra- and infrapyramidal blades clearly separate. Image z-stacks (30–35 images per stack; z-axis interval between consecutive frames: 1 μm) were oriented perpendicular to the longitudinal axis of the GCL. Image stacks belonging to each section were saved in the ics/ids (Image Cytometry Standard) file format and analyzed with the Fiji software (Image Processing and Analysis in Java, version 1.48s). All Prox1-positive cells were counted, including the following: DCX+/CB−, CB+/DCX−, DCX+/CB+ and DCX−/CB− which were standardized against the total number of Prox1-positive cells. Cells were counted in single images chosen from each z-stack and co-localization was determined by overlapping signals in each individual channel using Fiji. Images were edited with Fiji and Adobe Photoshop CS6 version 13.0 x64 for contrast, rotation and selection of region of interest. Quantifications were done with Microsoft Excel and GraphPad Prism 6. A monoexponential function fit was used to determine the average age at which 50% of Prox1-positive cells were DCX-positive as well as the age when 50% of Prox1-positive cells were CB-positive (i.e., t_50%Prox1_) for both, mice and rats using the following model: Y = (Y0 − Plateau) * exp (−K * X) + Plateau in GraphPad Prism 6. In addition, it was determined at what average age the half maximum decay of DCX expression was reached based on the first data point at P7 for both species (i.e., t_50%DCX_). Figures were prepared with Adobe Illustrator CS6 version 16.0.0.

### Statistical Analysis

Statistical analysis and data visualization were done with Microsoft Excel and GraphPad Prism 6. All statistical testing was performed with the two-way ANOVA followed by a *post hoc* Bonferroni test. Significance level was set to *P* < 0.05, denoted by an asterisk (*). Results are expressed as mean ± SEM.

## Results

**Figure 1:**
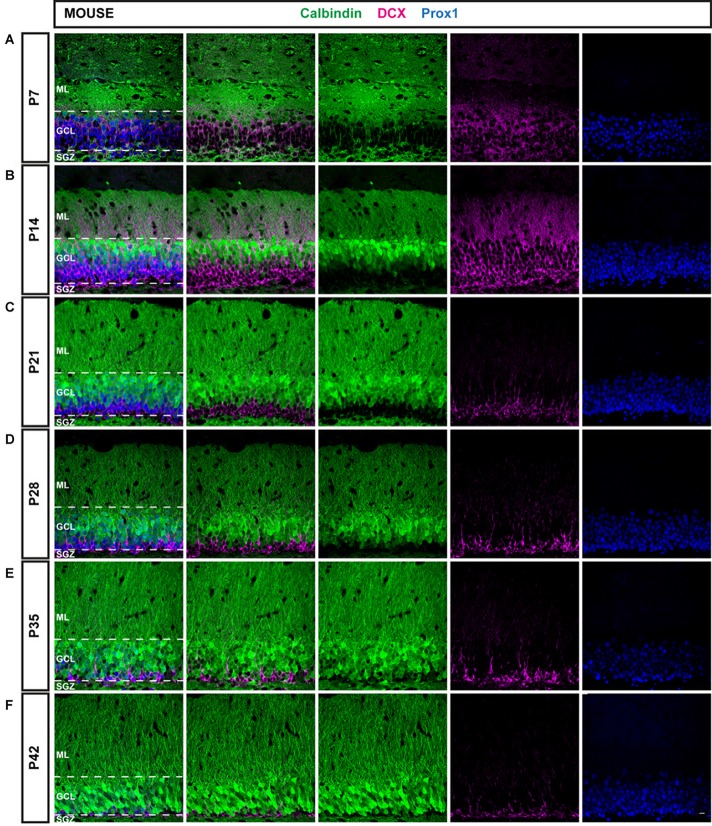
Immunohistochemical illustration of calbindin (CB), doublecortin (DCX), and prospero-related homeobox 1 (Prox1) distribution in the postnatal dentate gyrus (DG) of the mouse.** (A–F)** Immunostainings of the postnatal mouse DG show increasing maturation from P7 to P42, as the proportion of mature CB-positive cells (green) increased while the number of young, immature DCX-expressing cells (magenta) was reduced over time. The granule cell (GC) marker Prox1 (blue) was expressed in both mature and immature GCs at all time points. Scale bar: 10 μm.

**Figure 2:**
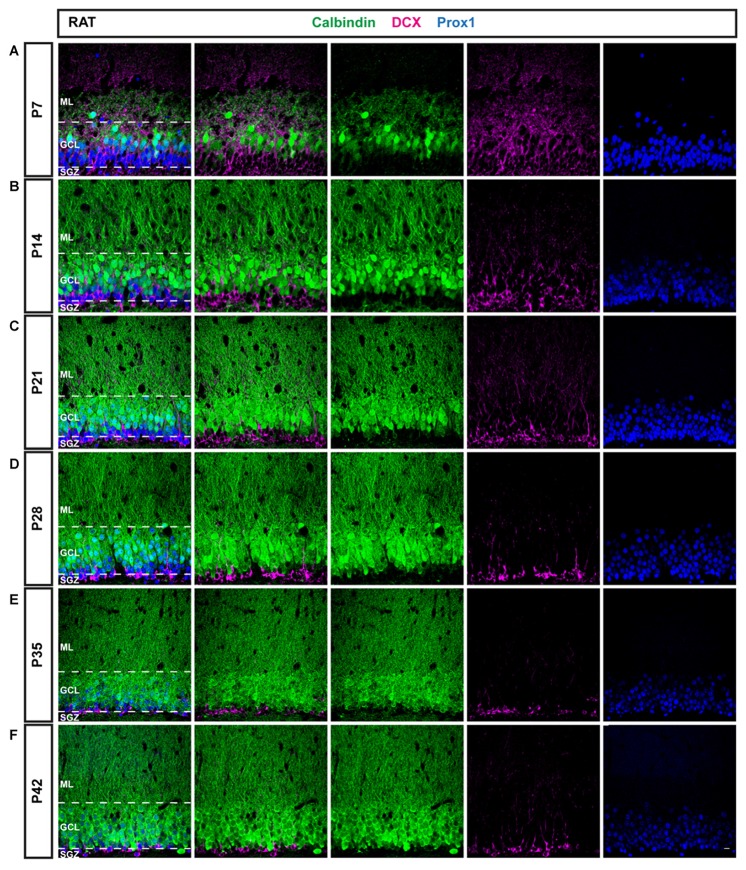
Immunohistochemical illustration of CB, DCX, and Prox1 distribution in the postnatal DG of the rat. **(A–F)** Immunostainings of the postnatal rat DG from P7 to P42 show increasing maturation, similarly as in the mouse. However, a larger proportion of GCs expressed CB (green) at early time points and the fraction of DCX-positive cells (magenta) decreased more rapidly than in the mouse, especially between P7 and P14. Prox1 (blue) was expressed in both mature and immature GCs at all time points. Scale bar: 10 μm.

The cytological composition of the DG in mice and rats was examined at different time points from the early postnatal period until animals reached sexual maturity, i.e., at P7, 14, 21, 28, 35 and 42 using immunohistochemistry. During this time period, the expression patterns of the immature neuronal marker DCX, a marker for mature neurons, CB, and a GC-specific marker, Prox1, were characterized in order to elucidate the course of maturation of the GC population and DG development during the early postnatal period in the two most commonly used rodent species, mice (Figure 1) and rats (Figure 2). Earlier postnatal time points were not considered as it was reported that the distribution of DCX is diffuse throughout the DG before P7, while from that point on, it is markedly expressed in the GCL (Nicola et al., 2015).

### The Distribution of DCX and CB in the Mouse DG between P7 and P42

**Figure 3:**
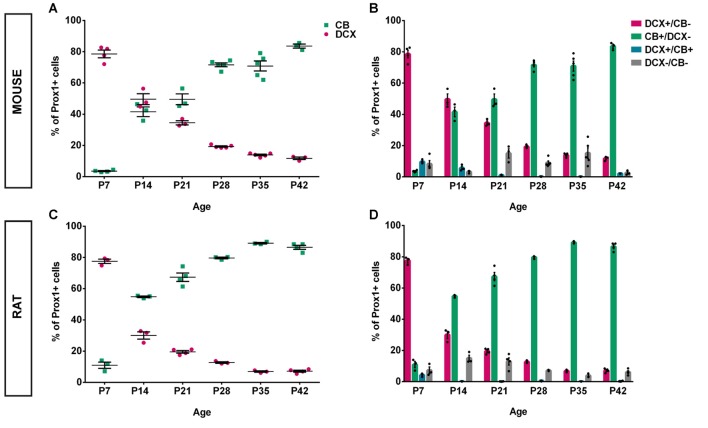
Quantification of CB and DCX expression in the postnatal mouse and rat DG over time. **(A)** In the mouse, at P7, nearly 80% of all Prox1-positive GCs expressed DCX while less than 4% were CB-positive. However, at P14, the number of DCX-positive GCs dropped to 50%, while 42% expressed CB. This represents the most drastic change during all time points that were examined. DCX expression continued to gradually decrease while the proportion of CB-expressing cells continued to increase until P42. **(B)** The relative distribution of DCX- and CB-expressing GCs shows a gradual shift toward maturity over time, particularly between P7 and P14. The number of triple-labeled cells that expressed Prox1 as well as DCX and CB constituted 9.62% at P7, less than 6% at P14 and P21, and between 0.11% and 2% from P28 to P42. In addition, Prox1-positive cells expressing neither maturity marker were noted. *n*_P7_ = 4; *n*_P14_ = 3; *n*_P21_ = 3; *n*_P28_ = 5; *n*_P35_ = 5; *n*_P42_ = 3. *n* = number of animals. **(C)** Similarly to the mouse DG, nearly 80% of all Prox1-positive GCs in the rat expressed DCX at P7 while 11% were CB-positive. At P14, the rat DG contained 30% DCX-positive and approximately 55% of CB-expressing GCs. Thus, the shift toward maturity between P7 and P14 was greater compared with the mouse. From then on, the proportion of DCX-positive GCs continued to decrease while the percentage of CB-expressing cells increased gradually until P28 after which there was only little change until P42. **(D)** The distributions of DCX and CB in GCs over time show a striking shift from immature to mature GCs between P7 and P14. The proportion of GCs expressing both DCX and CB was extremely low at all time points, with 4% at P7 and less than 0.6% at all other time points. The fraction of GCs not expressing either maturation marker constituted between 7%–15% at P7–21 and 4%–7% at P28–42. *n*_P7_ = 3; *n*_P14_ = 3; *n*_P21_ = 4; *n*_P28_ = 3; *n*_P35_ = 3; *n*_P42_ = 4. *n* = number of animals.

An overview of CB (green), DCX (magenta), and Prox1 (blue) expression in the mouse DG between P7 and P42 is presented in Figure 1. At P7, extensive DCX expression was observed throughout the GCL of the mouse DG, whereas CB-positive GCs were localized only in the very outer part of the GCL. Hence, the majority of Prox1-positive cells expressed DCX (78.57 ± 2.44% of all Prox1-positive cells) while only a small number of GCs was CB-positive (3.56 ± 0.33%; Figure 3A). In addition, a small proportion of Prox1-positive cells co-expressing both DCX and CB were counted (9.62 ± 0.57%) as well as GCs that were not labeled by either maturity marker (8.25 ± 2.19%; Figure 3B). Therefore, at an early postnatal time point, when the development of the DG is still ongoing and the establishment of the “adult” neurogenic niche in the SGZ has presumably begun (Nicola et al., 2015), the vast majority of dentate GCs was young and immature. At P14, when the adult neurogenic niche is established (Nicola et al., 2015), and animals begin to open their eyes and start to become active, still a majority of all GCs was DCX-positive (49.60 ± 3.49%) although a notably increased number of GCs expressed CB at this point (41.54 ± 3.08%; Figure 3A). Only a minor proportion of Prox1-positive cells expressed both DCX and CB (5.67 ± 1.12%) or neither maturity marker (3.19 ± 0.52%; Figure 3B). At P21, the distribution of immature and mature GCs shifted, as 34.47 ± 1.32% of GCs were DCX-positive, while the majority (49.52 ± 3.50%) expressed CB (Figure 3A). Only 1.01 ± 0.27% of all GCs were labeled with both DCX and CB, while 15.01 ± 2.98% exhibited neither of these two markers (Figure 3B). At P28, young, immature DCX-positive cells constituted a clear minority of all Prox1-positive cells (19.30 ± 0.42%) and were found in the SGZ and only the inner part of the GCL, whereas at this time point, CB expression was prominent throughout the middle and outer parts of the GCL (71.57 ± 1.23%; Figure 3A). The proportion of GCs expressing both DCX and CB decreased to 0.16 ± 0.05%, while 8.98 ± 1.18% of GCs didn’t express either maturation marker (Figure 3B). Thereafter, only little change in the marker distribution took place, as at P35 13.90 ± 0.51% of GCs were DCX-positive, 70.86 ± 3.20% expressed CB, 0.11 ± 0.04% were labeled with both markers, and 15.13 ± 3.37% exhibited neither DCX nor CB expression (Figures 3A,B). Similarly, at P42 11.75 ± 0.80% were DCX-positive, 83.61 ± 1.32% expressed only CB, 2.08 ± 0.02% exhibited both markers, and 2.55 ± 0.79% expressed neither (Figures 3A,B). These results demonstrate that the number of young, immature DCX-labeled GCs constituted the majority of all GCs in the mouse DG at early postnatal time points P7 and P14, but their number steadily decreased over time. In contrast, mature CB-positive GCs were rarely found in the 7-day old mouse, whereas their number increased substantially over the next several weeks until P28 when it did not change further.

### The Distribution of DCX and CB in the Rat DG between P7 and P42

To compare and contrast the developmental course of the dentate GC population between the mouse and the rat, the distribution of DCX and CB within Prox1-positive cells was examined in the rat DG between P7 and P42 as well (Figure 2). Similarly to the mouse DG, DCX expression was widespread in most parts of the rat GCL at P7 (77.54 ± 1.39%) while only a low number of CB-positive GCs was observed in the outer parts of the GCL (10.95 ± 2.02%; Figure 3C). Again, a small proportion of Prox1-positive cells co-localized with both, DCX and CB (4.24 ± 0.63), whereas a few GCs expressed neither marker (7.28 ± 2.13%; Figure 3D). However, at P14 already, DCX-positive GCs represented the minority (29.99 ± 2.33%) while there was a strong increase in the proportion of CB-positive GCs (54.81 ± 0.50%; Figure 3C). At this time point, 0.20 ± 0.05% of all GCs co-expressed both markers, and 15.00 ± 2.01% expressed neither (Figure 3D). At P21, the number of immature GCs decreased further, as 19.55 ± 0.87% were DCX-positive, while the percentage of CB-expressing GCs continued to increase (67.33 ± 2.69%; Figure 3C). Again, a very small number of GCs was labeled with both markers (0.13 ± 0.05%), and some GCs (12.99 ± 2.30%) exhibited neither (Figure 3D). At P28, DCX expression was observed in 12.72 ± 0.51% of all GCs, while the vast majority, 79.64 ± 0.55%, were CB-positive (Figure 3C). 0.52 ± 0.16% of all GCs co-expressed both maturity markers and 7.12 ± 0.22% exhibited neither (Figure 3D). At P35, a very low number of DCX-positive GCs was observed only in the SGZ and inner GCL (6.88 ± 0.47%), while most cells in the GCL exhibited CB expression and were thus mature (89.12 ± 0.44%; Figure 3C). Only a small number of GCs were labeled with both markers (0.14 ± 0.01%), or neither (3.86 ± 0.72%; Figure 3D). Finally, at P42 there was almost no change, as 7.16 ± 0.64% of Prox1-positive cells were DCX-positive, 86.47 ± 1.29% co-expressed CB, 0.26 ± 0.12% were labeled with both DCX and CB, and 6.11 ± 0.93% expressed neither (Figures 3C,D). The general expression patterns and changes in distribution of DCX and CB in the early postnatal rat DG over time were comparable to the mouse data. However, there were some important differences in the exact time course of these changes.

### The Postnatal Maturation Pattern Is Faster in Rats Compared to Mice

**Figure 4:**
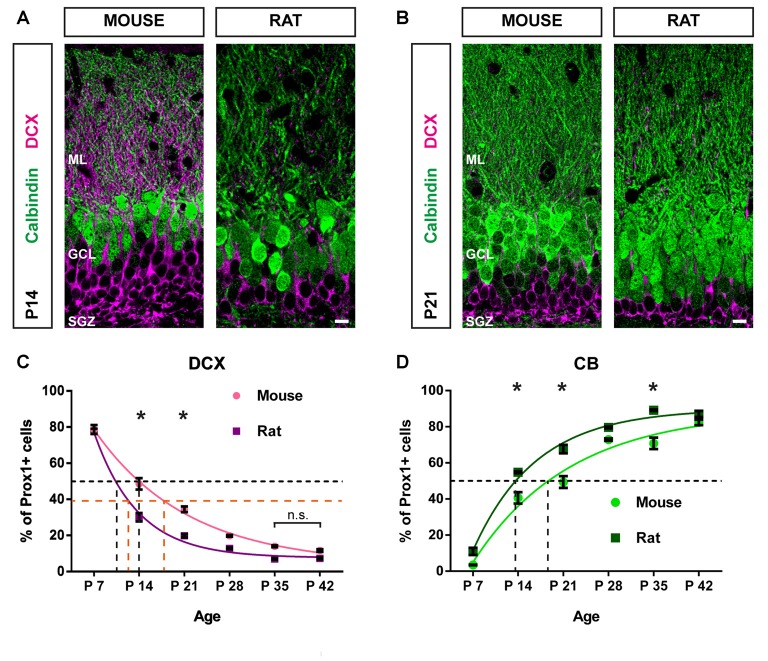
Postnatal maturation of the DG progresses faster in the rat compared with the mouse.** (A)** Immunohistochemical depiction of DCX- (magenta) and CB-expressing GCs (green) in the mouse and rat DG at P14 and **(B)** at P21. **(C)** The comparison of DCX-positive GC distribution between mice and rats from P7 to P42 reveals that the proportion of DCX-expressing cells decreased more rapidly in the rat. Significant differences in the number of DCX-positive cells between species were found on P14 (two-way ANOVA with Bonferroni correction *P* < 0.05) and P21. There were no significant differences in the proportion of DCX-positive cells between P35 and P42 within groups, indicating that a steady state of DCX expression has been reached in both, mice and rats. A monoexponential fit was used to determine the average age at which 50% of Prox1-positive cells were DCX-positive (i.e., t_50%Prox1_) in each species (black dashed lines). While for mice, t_50%Prox1_ was at 14 days, in the rat, it was at 10 days. Furthermore, the time to half maximum decay of DCX expression from the first data point at P7 was determined for each species (i.e., t_50%DCX_). In mice t_50%DCX_ was at 18 days, whereas in rats, t_50%DCX_ occurred at 12 days (orange dashed lines). **(D)** The proportion of CB-positive GCs increased more quickly in the rat compared with GCs in the mouse. Significant differences in the number of CB-positive cells between species were found on P14 (two-way ANOVA with Bonferroni correction *P* < 0.05), P21 and P35. In the mouse t_50%Prox1_ for Prox1-positive GCs expressing CB was 19 days, while in the rat, t_50%Prox1_ occurred at 14 days (black dashed lines). These findings suggest that GCs mature faster in the rat compared with the mouse. Mouse: *n*_P7_ = 4; *n*_P14_ = 3; *n*_P21_ = 3; *n*_P28_ = 5; *n*_P35_ = 5; *n*_P42_ = 3. Rat: *n*_P7_ = 3; *n*_P14_ = 3; *n*_P21_ = 4; *n*_P28_ = 3; *n*_P35_ = 3; *n*_P42_ = 4. *n* = number of animals. Error bars represent SEM. *P < 0.05. Scale bars: 10 μm.

In a direct comparison of the distribution patterns of immature GCs (DCX-positive) and mature GCs (CB-positive) in the postnatal rat and mouse DG, we could determine that the course of GC population maturation was faster in the rat (Figure 4). The proportion of DCX-positive GCs in the rat decreased significantly from P7 (77.54 ± 1.39%) to P14 (29.99 ± 2.33%, two-way ANOVA: time effect *F*_(5,31)_ = 643.9, *P* < 0.05, followed by *post hoc* Bonferroni’s test, *P* < 0.05), and between P14 and P21 (19.55 ± 0.87%, *P* < 0.05). In the mouse, the decrease in the percentage of immature GCs between P7 and P14 was less drastic, albeit highly significant (P7: 78.57 ± 2.44%; P14: 49.60 ± 3.49%, *P* < 0.05) as well as between P14 and P21 (34.47 ± 1.32%, *P* < 0.05), and between P21 and P28 (19.30 ± 0.42%, *P* < 0.05; Figure 4C). There were no significant differences in the proportion of DCX-positive cells between P35 and P42 within groups, which implies that a steady state of DCX expression has been reached in both, mice and rats (Figure 4C).

Significant differences in the proportion of DCX-expressing GCs between mice and rats were observed at P14 and P21. The mouse DG contained a significantly higher percentage of DCX-positive GCs at P14 (mouse: 49.60 ± 3.49%; rat: 29.99 ± 2.33%, two-way ANOVA: group effect *F*_(1,31)_ = 112.6, *P* < 0.05, followed by *post hoc* Bonferroni’s test, *P* < 0.05; group × time interaction: *F*_(5,31)_ = 10.54, *P* < 0.05) and P21 (mouse: 34.47 ± 1.32%; rat: 19.55 ± 0.87%, *P* < 0.05; Figures 4A–C). Next, we determined the average age at which 50% of all Prox1-positive cells co-expressed DCX (i.e., t_50%Prox1_) for each species by applying a monoexponential fit. In mice, t_50%Prox1_ was at 14 days, whereas in the rat, t_50%Prox1_ was at 10 days (black dashed lines in Figure 4C). Thus, the decrease of immature DCX-labeled GCs to 50% of the GC population occurred faster in the rat by approximately 4 days. Along the same lines, the time to half maximum decay of DCX expression from the first data point at P7 was determined for each species as well (i.e., t_50%DCX_). While in mice the time to half maximum decay was 18 days, in rats it occurred at 12 days, thus 6 days earlier (orange dashed lines in Figure 4C).

In addition, the increase in mature, CB-positive GCs was more rapid in the rat than in the mouse (Figure 4D). In the rat, the increase in CB-expressing GCs was significant between P7 (10.95 ± 2.02%) and P14 (54.81 ± 0.50%, two-way ANOVA: time effect *F*_(5,31)_ = 350.7, *P* < 0.05, followed by *post hoc* Bonferroni’s test, *P* < 0.05), between P14 and P21 (67.33 ± 2.69%, *P* < 0.05), and between P21 and P28 (79.64% ± 0.55, *P* < 0.05). In the mouse, the increase in the proportion of CB-expressing GCs was significant between P7 (3.56 ± 0.33%) and P14 (41.54 ± 3.08%, *P* < 0.05) as well as between P21 (49.52 ± 3.50%) and P28 (71.57 ± 1.23%, *P* < 0.05), and P35 (70.86 ± 3.20%) and P42 (83.61 ± 1.32, *P* < 0.05; Figure 4D).

A significantly higher proportion of GCs co-expressing CB was found in the rat DG at P14 (mouse: 41.54 ± 3.08%; rat: 54.81 ± 0.50%, two-way ANOVA: group effect *F*_(1,31)_ = 79.29, *P* < 0.05, followed by *post hoc* Bonferroni’s test, *P* < 0.05; group × time interaction: *F*_(5,31)_ = 4.065, *P* < 0.05), P21 (mouse: 49.52 ± 3.50%; rat: 67.33 ± 2.69%, *P* < 0.05), and P35 (mouse: 70.86 ± 3.20%; rat: 89.12 ± 0.44%, *P* < 0.05; Figure 4D). The average age at which 50% of all GCs were CB-positive in the mouse was 19 days, while in the rat, t_50%Prox1_ occurred at 14 days (black dashed lines in Figure 4D). Hence, the increase in the fraction of GCs co-expressing CB was faster by 5 days in the rat compared to the mouse.

These data indicate that the early postnatal maturation course of the dentate GC population is faster in the rat, as the rat DG contained a higher number of mature GCs and fewer young, immature GCs at P14 and P21 in comparison to the mouse. However, at P42, when the animals have transitioned into sexual maturity, the relative numbers of both mature and immature GCs were comparable between the two species.

## Discussion

This study presents an overview of postnatal development in the rodent DG. The time course of postnatal development and maturation of the hippocampal GC population was investigated based on the distribution patterns of the immature neuronal marker DCX and a marker for mature neurons, CB, at different time points from the early postnatal period until animals reached sexual maturity. As mice and rats represent the most widely used experimental mammals, postnatal maturation of the GC population was studied in both species and species-dependent differences were investigated.

At P7, the vast majority of neurons in both, rats and mice, were still young and immature, with almost 80% of all GCs expressing DCX. However, between P7 and P28, a drastic change toward the mature phenotype took place in both species, as the proportion of mature CB-positive GCs increased and the percentage of immature DCX-positive GCs dropped substantially (see Figure 3). In direct comparison, the shift toward a mature phenotype was considerably faster in rats and preceded GC population maturation in mice by 4–6 days. These results suggest that during the early postnatal period, the rat DG contains a higher proportion of matured GCs compared to the mouse. Interestingly, this is also the time frame during which the neurogenic niche in the putative SGZ is formed (Schlessinger et al., 1975; Altman and Bayer, 1990a; Hodge et al., 2013). In a recent study, Nicola et al. (2015) used a Nestin-GFP reporter mouse and additionally applied other markers including Prox1 and DCX to follow the development of the DG from E16.5 to P30. Confirming previous reports on the development of the DG in the rat (Altman and Bayer, 1990a) but employing modern markers, the mouse study shows that the differentiation of the SGZ as a separate entity begins around P7 and is completed by P14, as Ki67- and Nestin-positive cells are found only within the SGZ at this point (Nicola et al., 2015). These findings reflect prior data showing that GFAP-expressing progenitor cells with a neuronal phenotype were mainly found in the GCL, SGZ and hilus at P5 and were completely confined to the SGZ by P14 (Seki et al., 2014). In addition, it was reported that the distribution of DCX in the DG is diffuse before P7, and at P7, it is widely expressed in the GCL (Nicola et al., 2015). This is consistent with our findings of a majority of 80% GCs being DCX-positive in mice and rats at P7 and becoming more confined to the inner parts of the GCL and the SGZ over time. Together, these data suggest that from the second postnatal week on, neurogenesis is a continuous process localized in the established postnatal subgranular neurogenic niche.

In this study we investigated the maturation progress of the postnatal GC population using the well-established neuronal maturation markers DCX and CB rather than birth-dating of individual GCs. Though this method does not indicate the exact age of each GC, the expression of DCX and/or CB corresponds to the maturation state of the GC population and is thus a valid approach for the study of DG maturation, even though individual GCs may not follow the same time course. Studies applying mitotic markers such as BrdU revealed that the expression of DCX and CB closely correlate with the age of newborn GCs, at least in adult mice and rats (Snyder et al., 2009; Jungenitz et al., 2014). Furthermore, the switch from DCX to CB occurs rapidly, resulting in only a small percentage of BrdU-labeled newborn GCs expressing both DCX and CB in the adult rodent (Snyder et al., 2009; Jungenitz et al., 2014). This indicates a close time-dependent maturation of the GC population which is reflected by the two markers. In the present study we show that the shift from DCX to CB in postnatal GCs is also very sharp but occurs considerably faster in the population of labeled GCs in rats compared to mice. Even though the largest shift toward maturation occurred between P7 and P14 in both species, it took an additional week in the mouse for the majority of GCs to reach maturity and DCX expression to drop under 50%. Only by the sixth week of postnatal development were the expression patterns of maturity markers comparable between the two species (Figure 4). These results reveal important differences between mice and rats that must be considered when comparing findings of developmental studies that were performed in different rodent species.

The fraction of postnatal DCX-positive cells is not only affected by neurons maturing and leaving this cell pool but also by the number of newly formed cells that replenish the DCX-positive cell pool. Since our data reflect changes of the total DCX-positive cell pool and not changes of individual neurons, species-differences in postnatal proliferation need to be considered. In fact, previous work has revealed differences in GC proliferation between mice and rats. In classical autoradiographic studies of mitotic markers, differences in the time of GC origin were demonstrated between the two species: in the mouse, first GCs are generated as early as E10 whereas in the rat, GC production begins around E16 (Angevine, 1965; Altman and Bayer, 1990b). Furthermore, the peak of DG cell proliferation takes place around birth (P1) in mice (Angevine, 1965) whereas in rats, DG maximal cell genesis in the so-called tertiary matrix occurs mainly postnatally and peaks between P5 and P8 (Schlessinger et al., 1975). In the current study we show that the proportion of DCX-expressing cells decreased to 30% at P14 in rats, while in the mouse, almost half of all GCs were still DCX-positive at this time point. Considering the delayed embryonic and extended postnatal proliferation time in rats, our findings came as a surprise, since we would have predicted that the shift from DCX to CB would mirror the proliferation peaks and occur later in rats. The opposite is the case, indicating that in spite of the late proliferation peak in rats, the young GC population transitions more quickly to a mature state in rats than in mice. This pattern of relative marker distribution changes persisted until P21 whereby rats were 4–6 days ahead of mice in relation to the course of maturation (see Figure 4).

Important species-dependent differences were also found during the process of adult neurogenesis (Snyder et al., 2009). Snyder et al. (2009) showed that adult-born GCs in the rat were more abundant, more likely to survive and become activated during learning tasks, and matured quicker than mouse adult-born GCs. In contrast, significantly more newborn neurons continued to express DCX in the mouse than in the rat. Overall, the proportion of DCX-positive cells in the rat was decreasing more rapidly by 1–2 weeks (Snyder et al., 2009). Recently, Cahill et al. (2017) reported that GCs labeled with BrdU at P6 in rats showed a similar time course of DCX expression as young adult-born (8–9 weeks) GCs, with a rapid decrease of DCX between the second and the third week post BrdU labeling. In addition, patterns of IEG expression suggest that postnatally born neurons mature even faster than adult-born neurons in the rat. Interestingly, in contrast to adult-born GCs, postnatally born (P6) neurons in the rat largely survive during the first weeks, but show delayed cell death between 2 and 6 months of age (Dayer et al., 2003; Cahill et al., 2017). Thus, postnatally born GCs may contribute considerably and with unique forms of plasticity to hippocampal function (Cahill et al., 2017). The findings of the current work in postnatal and juvenile mice and rats are in line with these data, as we show that GC maturation on the population level was quicker in rats compared with mice, suggesting that similar species differences in developmental mechanisms are at play in the postnatal as in the adult DG.

The postnatal development of the DG in both the mouse and the rat, appears to follow a clear maturation course that displays the establishment of an outside-in pattern of the GCL with older, mature GCs positioned in the GCL part close to the ML, while young and newborn cells continue to be added to the inner parts of the GCL, closer to the hilus (Altman and Bayer, 1990a; Mathews et al., 2010; Radic et al., 2015). As the DG develops, the production and presence of newborn, immature GCs gradually becomes confined to the innermost part of the GCL. The postnatal hilar zone of intrahippocampal neurogenesis constitutes the SGZ where neurogenesis continues as an ongoing process from the postnatal to the adult state (Altman and Bayer, 1990a; Nicola et al., 2015).

Besides the species- and age-dependent differences in the time course of GC population maturation, the distribution patterns of young and old GCs from the early postnatal (P7) to the young adult phase (P42) show considerable similarities in mice and rats and follow a clear logic of a deceleration of maturation. Thus, the postnatal phase can be regarded as a valuable correlate to study processes of neurogenesis in a developing and maturing neural network. Indeed, postnatal neurogenesis can be effectively studied in organotypic hippocampal slice cultures (OTCs) of the rat prepared at P5 using the same neuronal maturation markers DCX and CB as well as retroviral labeling of newborn GCs (Radic et al., 2017). The distribution of DCX and CB at day *in vitro* (DIV) 7 to DIV 28 in OTCs exhibited a similar pattern to our *in vivo* findings (Radic et al., 2017). In conclusion, the postnatal development of the DG appears to be an important phase of preservation of neurogenesis with a species-dependent deceleration of GC maturation that can be used for future studies on regulation of ongoing neurogenesis in a maturing neural network, e.g., in postnatal organotypic hippocampal cultures.

## Author Contributions

TR, TJ and SWS: conceived and designed the experiments; TR, LF and AV: performed the experiments; TR, LF, AV, TJ, TD and SWS: analyzed and discussed the data; TD and SWS: contributed reagents/materials/analysis tools; TR and SWS: wrote the article. All authors reviewed the manuscript.

## Conflict of Interest Statement

The authors declare that the research was conducted in the absence of any commercial or financial relationships that could be construed as a potential conflict of interest.
